# Association of different positive end-expiratory pressure selection strategies with all-cause mortality in adult patients with acute respiratory distress syndrome

**DOI:** 10.1186/s13643-021-01766-7

**Published:** 2021-08-12

**Authors:** Manuel Tisminetzky, Jose Dianti, Bruno L. Ferreyro, Federico Angriman, Lorenzo Del Sorbo, Sachin Sud, Daniel Talmor, Eddy Fan, Niall D. Ferguson, Ary Serpa Neto, Neill K. J. Adhikari, Ewan C. Goligher

**Affiliations:** 1 University Health Network/Sinai Health System, University of Toronto, Toronto, Canada; 2 Interdepartmental Division of Critical Care Medicine, University of Toronto, Toronto, Canada; 3 Institute of Health Policy, Management and Evaluation, Dalla Lana School of Public Health, University of Toronto, Toronto, Canada; 4 Department of Critical Care Medicine, Sunnybrook Health Sciences Centre, Toronto, Canada; 5 Institute of Medical Science, University of Toronto, Toronto, Canada; 6 Division of Respirology and Critical Care Medicine, Toronto General Hospital, 585 University Ave. 11-PMB, Room 192, Toronto, ON M5G 2N2 Canada; 7 Institute for Better Health and Critical Care, Department of Medicine, Trillium Health Partners, Mississauga, Canada; 8 Department of Anesthesia, Pain, Medicine and Critical Care, Beth Israel Deaconess Medical Center, Harvard Medical School, Boston, USA; 9 Hospital Israelita Albert Einstein, São Paulo, Brazil; 10 Toronto General Hospital Research Institute, Toronto, ON Canada

**Keywords:** ARDS, Acute respiratory distress syndrome, PEEP, Positive end-expiratory pressure, Hypoxemic respiratory failure, Barotrauma, Lung recruitment maneuver

## Abstract

**Background:**

The acute respiratory distress syndrome (ARDS) has high morbidity and mortality. Positive end-expiratory pressure (PEEP) is commonly used in patients with ARDS but the best method to select the optimal PEEP level and reduce all-cause mortality is unclear. The primary objective of this network meta-analysis is to summarize the available evidence and to compare the effect of different PEEP selection strategies on all-cause mortality in adult patients with ARDS.

**Methods:**

We will search MEDLINE, Cochrane Central Register of Controlled Trials, PubMed, EMBASE, and LILACS from inception onwards for randomized controlled trials assessing the effect of PEEP selection strategies in adult patients with moderate to severe ARDS. We will exclude studies that did not use a lung-protective ventilation approach as part of the comparator or intervention strategy. The primary outcome will be all-cause mortality (at the longest available follow-up and up to 90 days). Secondary outcomes will include barotrauma, ventilator-free days, intensive care unit and hospital length of stay, and changes in oxygenation. Two reviewers will independently screen all citations, full-text articles, and extract study-data. We will assess the risk of bias for each of the outcomes using version 2 of the Cochrane risk of bias tool for randomized controlled trials. If feasible, Bayesian network meta-analyses will be conducted to obtain pooled estimates of all potential head-to-head comparisons. We will report pairwise and network meta-analysis treatment effect estimates as risk ratios and risk differences, together with the associated 95% credible intervals. We will assess certainty in effect estimates using GRADE methodology.

**Discussion:**

The present study will inform clinical decision-making for adult patients with ARDS and will improve our understanding of the limitations of the available literature assessing PEEP selection strategies. Finally, this information may also inform the design of future randomized trials, including the selection of interventions, comparators, and predictive enrichment strategies.

**Trial registration:**

PROSPERO 2020 CRD42020193302.

**Supplementary Information:**

The online version contains supplementary material available at 10.1186/s13643-021-01766-7.

## Background

The acute respiratory distress syndrome (ARDS) is a life-threatening form of noncardiogenic pulmonary edema that leads to hypoxemia, hypercapnia, and a decrease in lung compliance, due to a direct or indirect insult to the lungs [1–3]. Despite 50 years of research, hospital mortality in patients with ARDS remains as high as 40% [4]. Since the initial description of this syndrome, positive end-expiratory pressure (PEEP) has been used to improve ventilation-perfusion matching and oxygenation by increasing the number of alveoli available for gas exchange, and to reduce the risk of ventilator-induced lung injury (VILI) [5, 6].

Mechanical ventilation may cause or exacerbate acute lung injury by applying excessive stress and strain to the lung parenchyma (i.e., *volutrauma*) and by inducing cyclic opening and closing of lung units (i.e., *atelectrauma*) [7, 8]. This injury further reduces the volume of the aerated lung, increasing stress and strain in the remaining aerated lung regions [6]. Setting adequate levels of PEEP may prevent lung de-recruitment and cyclic opening and closing, potentially preventing VILI as shown in experimental models [9, 10]. However, increased PEEP may also lead to increased alveolar dead space and alveolar overdistention [11, 12]. Multiple PEEP selection strategies have been described, each based on different physiologic considerations. It is uncertain, however, the degree to which specific PEEP selection strategies are more effective in preventing ventilator-induced lung injury and reducing mortality in adult patients with ARDS [13].

### PEEP titration strategies based on oxygenation

Several previous studies have addressed this question without consistent findings. In 2004, a landmark trial compared ventilation with a low PEEP:FiO_2_ table to a higher PEEP:FiO_2_ table [14]. Although PaO_2_:FiO_2_ during the first 7 days was higher in the high PEEP:FiO_2_ table group, no significant difference in mortality rates was found. Mercat et al. conducted a randomized clinical trial that compared a minimal distention strategy (PEEP set at 5–9 cm H_2_O) to a high PEEP strategy (level of PEEP set to reach a plateau pressure of 28–30 cm H_2_O) [15]. The strategy was defined by a PEEP level aiming to increase alveolar recruitment while limiting hyperinflation but failed to significantly reduce mortality. However, a statistically significant reduction in the duration of both mechanical ventilation and organ failure was evident.

### PEEP titration strategies based on esophageal manometry

An alternative approach of titrating PEEP based on measurements of esophageal pressure has also been evaluated by Talmor and colleagues [16, 17]. Specifically, in the esophageal-pressure-guided group, PEEP was set to achieve an end-expiratory transpulmonary pressure between 0 and 10 cm H_2_O, while in the control group PEEP was adjusted according to the lower PEEP:FiO_2_ table used by the aforementioned ARDS Network study [16]. The trial was underpowered to show a difference in mortality between the two groups, but oxygenation and compliance were significantly increased in the esophageal pressure-guided group. However, the mortality benefit was not demonstrated in a subsequent much larger trial reported by Beitler et al. [18].

### PEEP titration strategies based on lung mechanics or following recruitment

Pintado and colleagues published a randomized controlled trial testing the hypothesis that setting PEEP based on the highest compliance compared to setting PEEP based on the ARDS Network study lower PEEP:FiO_2_ table would improve oxygenation [19]. The authors found no significant improvement in oxygenation and the study was not powered to assess mortality differences between groups. Also using static compliance to titrate PEEP, Kacmarek et al. proposed an open lung approach by which a recruitment maneuver (pressure control ventilation to a peak pressure between 50 and 60 cm H_2_O and PEEP 35 to 45 cm H_2_O depending on patient’s response) was followed by a decremental PEEP trial, and PEEP was set 3 cm H_2_0 above the maximum compliance [20]. In the control group, PEEP was set according to the low ARDS Network protocol table. The study did not find a significant difference in all-cause mortality at 60 days. Finally, the Alveolar Recruitment for Acute Respiratory Distress Syndrome Trial used a similar approach to determine if lung recruitment associated with PEEP titration based on the best respiratory system compliance decreased 28-days mortality [12]. The authors found that this strategy was associated with significantly higher 28-day and 6-month mortality rates, and a higher rate of barotrauma compared to the low PEEP:FiO_2_ table.

### The need for a network meta-analysis

Limiting tidal volume (*V*_T_) and plateau pressure are the key components of lung-protective ventilation [3], and although PEEP has been used since the first description of ARDS, it remains unclear which is the best method to adjust it in order to improve patients’ outcomes [7, 21, 22]. In clinical practice, and as a result of the multiple PEEP selection strategies used in randomized trials to date, different strategies are applied and preferences are largely based on local practice and physician’s expertise [4, 23]. Hence, there is a need to define which strategy for PEEP selection, if any, is best at improving clinically relevant outcomes in critically ill adults with ARDS. Several meta-analyses have evaluated whether high PEEP (with or without a lung recruitment maneuver) improves clinically relevant outcomes when compared to a lower PEEP strategy in adult patients with ARDS [24–27]. However, these have been hindered by several limitations, most notably the lack of differentiation of the distinct physiologic mechanisms used for PEEP titration, generally considering high PEEP as a single strategy rather than as multiple discrete interventions.

To this end, network meta-analysis represents a tool to generate pooled effect estimates for comparisons between multiple treatments and comparators [28]. Specifically, network meta-analysis incorporates estimates from both direct and indirect comparisons to estimate treatment effects where head-to-head comparisons are insufficient. This not only provides new information (e.g., estimates for comparisons that are not available based on direct evidence) but also reduces the imprecision in pooled treatment effects even when head-to-head comparisons are available. To our knowledge, no previous network meta-analyses have been conducted to compare the relative effectiveness of multiple PEEP selection strategies examined in randomized controlled trials that included adult patients with ARDS.

The primary objective of this study is to assess the association between PEEP selection strategies and all-cause mortality in adult patients with moderate to severe ARDS. The secondary objective is to assess the association between different PEEP selection strategies and barotrauma, response in oxygenation, ventilator-free days and intensive care unit and hospital length of stay in adult patients with ARDS.

## Methods

This protocol is reported in accordance with the Preferred Reporting Items for Systematic Review and Meta-Analysis Protocols 2015 statement (PRISMA-P) [29] (see checklist in Additional file 1), and its extension for network meta-analysis [30]. This protocol is registered in the International Prospective Register of Systematic Reviews (PROSPERO, CRD42020193302).

### Criteria for included studies

#### Study design and publication types

We will only include randomized controlled trials. Cross-over trials will be excluded.

#### Participants and settings

Studies will be selected according to the following criteria: study design, participants and settings, interventions and comparators, and outcomes of interest. We will include all randomized controlled trials that enrolled adult patients (18 years of age or older) with ARDS, defined by either the American-European Consensus Conference or the Berlin criteria [31, 32]. Trials will be eligible for inclusion if a lung protective ventilation strategy (i.e., *V*_T_ less than 6–8 ml/kg of predicted body weight (PBW); plateau pressure ≤ 30 cm H_2_O) was employed in both arms. Studies that employed ventilation strategies with *V*_T_ greater than 6–8 ml/kg PBW in either arm will be excluded. This exclusion is justified by the proven superiority and widespread use of low *V*_T_ ventilation strategies for ARDS [33]. Specifically, including studies that compared a lung-protective ventilation approach with a specific PEEP titration strategy to a non-protective *V*_T_ strategy (i.e., *V*_T_ > 8 ml/kg PBW) would render the resulting effect confounded by the effects of both lung-protective ventilation and PEEP. For the primary analysis, we will only include data on patients with moderate to severe ARDS as defined by PaO_2_:FIO_2_ < 200 [31].

#### Interventions and comparators

All interventions considered in this review are PEEP selection strategies that either result in higher PEEP levels or have a different physiological basis for PEEP selection compared to the control strategy in each study. First, we will consider strategies that adjust PEEP based on FiO_2_ levels. The traditional lower PEEP strategy employs a PEEP:FiO_2_ table as used by the Acute Respiratory Distress Syndrome Network [34]. Of note, the low PEEP:FiO_2_ table is the most frequently used strategy in control groups for trials assessing PEEP selection strategies. The higher PEEP strategy will refer to tables with higher PEEP for each FiO_2_ level than the one originally used in the ARDS Network trials [14]. The higher PEEP strategy will also be comprised, for the primary analysis, of additional approaches to select higher PEEP such as those based on lung mechanics (i.e., targeting a specific plateau pressure or the best static compliance) or other measurements reflective of lung recruitment (e.g., lung ultrasound). Second, we will identify a separate category of trials that employed a lung recruitment maneuver (i.e., open lung strategies). For this review, a lung recruitment maneuver will be defined as any form of transient elevation in mean airway pressure undertaken to reduce atelectasis (which may be either brief or prolonged) [25]. Third, studies employing esophageal manometry—a technique that guides PEEP selection according to transpulmonary pressure (*P*_L_)—will be considered a separate strategy. Esophageal manometry techniques may increase PEEP in some patients and lower PEEP in others; consequently, they represent a qualitatively different category than other higher PEEP titration strategies. Table 1 provides a description of the potential PEEP selection strategies that will be included in our network meta-analysis.

**Table 1:** Different available positive-end-expiratory pressure (PEEP) titration strategies for adult patients with acute respiratory distress syndrome

Strategy	Landmark study	Description
Low PEEP:FiO_2_ table	Brower, 2004 Meade, 2008	PEEP depends on level of required oxygenation (i.e., 10 cmH_2_O for a required FiO_2_ = 60%)^a^
High PEEP:FiO_2_ table	Brower, 2004	PEEP depends on level of required oxygenation (i.e., 16 cmH_2_O for a required FiO_2_ = 40%)
Higher PEEP based on lung mechanics	Mercat, 2008 Pintado, 2013 Salem, 2020	PEEP depends on a specific measure: - Plateau pressure < 30 cmH_2_O - Highest static compliance - PEEP depends on the classification of patients using lung ultrasound
PEEP titration based on esophageal manometry	Talmor, 2008 Beitler, 2019	PEEP depends on the transpulmonary pressure measured by esophageal manometry
PEEP titration following a recruitment maneuver^b^	Meade, 2008 Cavalcanti, 2017	PEEP titration (either based on FiO_2_ or lung mechanics) following a recruitment maneuver

^a^This arm will also include low PEEP strategies without a specific PEEP:FiO_2_ table such in Mercat 2008

^b^Different recruitment maneuvers will be considered (e.g., brief maneuvers including higher pressures for less than 60 s vs. the use of staircase maneuvers)

### Outcomes measures

The primary outcome of this study will be all-cause mortality, defined as the longest available in the first 90 days after randomization. Whenever necessary, 90-day mortality will be substituted by 60-day mortality, hospital mortality, 28- or 30-day mortality, or ICU mortality, in decreasing order of preference. The secondary outcomes will include barotrauma (defined as radiographic pneumothorax or chest tube insertion), oxygenation response (defined as an increase in oxygenation in response to a change from the initial PEEP level), ventilator-free days to day 28 [35], and length of intensive care unit and hospital stay.

### Information sources and search strategy

The following five electronic bibliographic databases will be searched using a comprehensive search strategy developed by an information specialist: (1) MEDLINE, (2) Cochrane Central Register of Controlled Trials, (3) PubMed (non-MEDLINE records only), (4) EMBASE, and (5) LILACS. A draft search strategy for MEDLINE is provided in Additional file 2. Databases will be searched from inception onwards, with no language restrictions. We will manage all references and duplicates using EndNote X8 citation management software.

### Study selection

Two reviewers (JD, MT, or FA) will independently screen the titles and abstracts retrieved from the search strategy and the additional sources in order to identify those meeting the mentioned eligibility criteria. Subsequently, we will obtain full texts of the articles meeting these pre-specified criteria and review again in a second stage. Any disagreement between the reviewers will be discussed and referred to final decision by a third investigator (ECG).

### Data extraction

Two reviewers (JD, MT, or FA) will perform data extraction independently in a preformatted form. Any differences will be resolved by consensus or discussion with a third author (ECG). Abstracted data will include study characteristics (trial design, size, and funding source), patients’ characteristics (age, sex, cause, and severity of ARDS), details of the interventions (type of PEEP selection strategy, PEEP levels in the first 7 days and adjunctive interventions), duration of follow-up, publication status, and outcome data for each endpoint of interest.

### Risk of bias assessment

Two reviewers (JD, MT, or FA) will independently assess the risk of bias, at the study level, using the version 2 of the Cochrane risk of bias tool for randomized trials (RoB 2) [36]. The following domains will be assessed: (1) randomization process, (2) deviations from the intended interventions, (3) missing outcome data, (4) measurement of the outcome, (5) selection of the reported result, and (6) overall risk of bias. Overall rating will be incorporated in the data synthesis as one of the key domains in the GRADE assessment (see below).

### Patient and public involvement

This research will be done without patient involvement. Patients will not be invited to comment on the protocol design nor consulted to develop patient-relevant outcomes.

### Data synthesis and analysis

We will summarize the included studies based on study and patient characteristics, outcome measures, and risk of bias. If feasible, we will perform a series of pairwise meta-analyses with a random-effects model, followed by a network meta-analysis using a Bayesian framework to derive head-to-head treatment effect estimates comparing all interventions. We will graphically represent the evidence using a network plot that incorporates nodes as treatment strategies, with connections representing direct head-to-head comparisons. Network geometry will thus show nodes as interventions and each head-to-head direct comparison as lines connecting these nodes. The size of the nodes will be proportional to the number of participants in each node. The thickness of the connecting line will be proportional to the number of randomized clinical trials in each comparison (depicted by the number beside line connecting nodes). We will depict the total number of participants by treatment strategy. In detail, we will also depict the total number of nodes, number of edges, common comparators, and average degree.

Analyses will be based on Markov chain Monte Carlo methods using minimally informative treatment effect estimates, and informative prior distributions for heterogeneity estimates derived from external evidence for each of the study outcomes [37]. Specifically, for all-cause mortality, the parameters of the predictive log-normal distribution for heterogeneity used as prior information will be μ =  − 3.50 and σ = 1.26. These prior distributions for heterogeneity have been previously derived for binary outcomes for a range of specific research settings by Turner et al. [37]. We will report pairwise and network treatment effect estimates as risk ratios and risk differences, estimating summary treatment effect estimates from the median and corresponding 95% credible intervals (CrIs) from the 2.5th and 97.5th percentile of the posterior distribution. For continuous outcomes, we will report mean differences with corresponding 95% Crls. We will also rank interventions according to their apparent effectiveness using the GRADE approach (see below).

We will quantify heterogeneity in treatment effects between studies using the posterior distribution τ. Inconsistency (incoherence) between direct and indirect comparisons will be estimated using the node-splitting approach [38]. We will visually assess model convergence using the Brooks-Gelman-Rubin diagnostic, trace plots, and auto-correlation plots. We will assess the goodness-of-fit of our final models by comparing the mean residual deviance with the number of contributing data points. We will perform all analyses in R v3.6 (packages meta, gemtc, pcnetmeta) using Just Another Gibbs Sampler (JAGS). Code and data will be made available upon request to the corresponding author.

### Assessment of publication bias

We will assess for the presence of publication bias by examining the comparison-adjusted funnel plot. We will examine the shape of the funnel plot and assume that there is a risk of publication bias if its shape is asymmetrical. We will formally test for asymmetry in the funnel plot by performing Beggs’s test [39].

### Additional analyses

Several a priori sensitivity analyses will be performed. First, to assess the potential impact of individual study quality in the observed effect estimates, a sensitivity analysis excluding studies with high risk of bias will be performed. Second, to show the impact of varying prior beliefs on the effect of a higher PEEP with lung recruitment maneuvers, we will update the network effect estimates for this group compared to the other categories using enthusiastic, skeptical, and pessimistic priors. Third, a sensitivity analysis recategorizing the strategies using a different and more granular grouping criteria will be performed. To this end, the higher PEEP category will be further divided into (1) studies that used a PEEP:FiO2 table or studies that used a plateau pressure limit (below 28–30 cm H_2_O), (2) studies that used best respiratory system compliance, and (3) studies that used lung ultrasound. Fourth, we will refit our network estimates using non-informative priors for the heterogeneity.

### Grading of recommendations

We will rate the certainty of each direct, indirect, and network estimates based on the approach suggested by the Grading of Recommendations Assessment, Development and Evaluation (GRADE) Working Group [40].

### Assessments of certainty in estimates of direct comparisons

We will use GRADE guidance to determine whether to rate down confidence for risk of bias, publication bias, and inconsistency for the body of evidence bearing on each direct estimate. In accordance with the GRADE approach for network meta-analysis [41], we will not rate down confidence for imprecision for direct comparisons. Instead, we will use GRADE guidance to determine whether to rate down the network comparison for imprecision (see below).

### Assessments of certainty in indirect comparisons

Indirect comparisons infer the relative effects of two interventions based on comparisons with other interventions. We will call them first order loops when the comparisons involve only a single additional intervention and higher order loops when comparisons involve two or more additional interventions. Confidence assessment for any given indirect comparison will focus on the associated first order loop with the lowest variance (i.e., the loop that contributes most to the estimate of effect). The confidence in estimates will be the lower of the confidence in each of the contributing direct comparisons. For instance, if A versus B warrants high confidence and B versus C warrants moderate confidence, we will judge the indirect estimate of A versus C as warranting moderate confidence. We may further rate down confidence in the indirect comparisons of the A versus B and B versus C comparison trials enrolled substantially different patients, or if the B intervention was implemented very differently (intransitivity).

### Assessments of confidence in the network estimates of effect

The judgment of confidence in the network estimate for any paired comparison will be the most dominant of the contributing direct and indirect comparisons. We will then rate down for serious imprecision or, if we find important differences between the direct and indirect estimates, for incoherence.

### Rating of interventions using GRADE approach

Because SUCRA and probabilistic ranking methods can generate erroneous inferences, we will use, in addition, the GRADE approach to rate interventions in the network [42]. First, we will choose lower PEEP as the reference strategy because it will likely be the most connected strategy (i.e., the most common comparator) in the network and because it is the PEEP level that most closely resembles clinical practice. Second, we will identify strategies that are at least ten times more likely to be beneficial than harmful compared against the reference, which we will categorize as likely superior. This threshold is based on the notion that changing PEEP levels represents a simple bedside procedure with no additional cost, so evidentiary burden required for action should be lower than for other more complex interventions used for the management of patients with ARDS such as extracorporeal life support. Next, we will repeat this procedure looking for strategies that are at least 10 times more likely to be beneficial than harmful when compared to at least one other effective strategy. Finally, we divided these categories into two groups according to certainty of evidence (high and moderate certainty vs. low and very low certainty). We will then check the accuracy of the categorizations by examining the associated SUCRA values.

Finally, protocol deviations from our PROSPERO registration and present protocol will be reported in a systematic fashion giving detailed explanations and reasoning.

## Discussion

The optimal PEEP selection strategy for adult patients with ARDS remains unknown. This Bayesian network meta-analysis will summarize the direct and indirect evidence on the efficacy of different available PEEP selection strategies to reduce all-cause mortality in adult patients with ARDS.

The present study may present several limitations, both at the individual-study and pooled levels. First, given the nature of the intervention at hand, individual studies are not expected to be blinded which may introduce bias mainly through the differential use of cointerventions. Second, we expect that individual studies included varying degrees of illness severity, thus representing a potential source of treatment heterogeneity. Finally, we will not have access to individual patient data, thus precluding us from further understanding the individual determinants for an optimal PEEP selection strategy.

Overall, this study may help identify, on average, an optimal PEEP selection strategy, adding new information to help clinicians during the daily decision-making process when caring for adult patients with ARDS. Furthermore, this information may also inform the design of future randomized controlled trials and aid in the identification of adequate interventions.

## Supplementary Information

**Additional file 1**. PRISMA-P 2015 Checklist.

**Additional file 2**. Search Strategies.

## Data Availability

Data sharing is not applicable to this article as no data set were generated or analyzed during the current protocol.

## Abbreviations

ARDS: Acute respiratory distress syndrome
PEEP: Positive end-expiratory pressure
VILI: Ventilator-induced lung injury
FiO2: Fraction of inspired oxygen
*V*_T_: Tidal volume
PBW: Predicted body weight
NMA: Network meta-analysis
SR: Systematic review
