# Supplementary material for: Association of different positive end-expiratory pressure selection strategies with all-cause mortality in adult patients with acute respiratory distress syndrome
Source: Syst Rev. 2021 Aug 12;10:225. doi: 10.1186/s13643-021-01766-7 (PMC8357961; doi:10.1186/s13643-021-01766-7)
Supplement: Supplementary file 2 — Additional file 2. Search Strategies. [file 13643_2021_1766_MOESM2_ESM.docx]

**Additional file 2.** Search Strategies

The following databases will be searched from inception, via the Ovid search interface: Medline, Medline In-Process/ePubs (daily), Embase, Cochrane Central Register of Controlled Trials. Lastly, the LILACS (Latin-American and Caribbean System on Health Sciences Information, BIREME - PAHO - WHO) database was searched.

The searching process will follow the Cochrane Handbook and the Cochrane Methodological Expectations of Cochrane Intervention Reviews (MECIR) for conducting the search, the PRISMA guideline for reporting the search, and the PRESS guideline for peer-reviewing the search strategies drawing on the PRESS 2015 Guideline Evidence-Based Checklist to avoid potential search error.

1) Medline

| **#** | **Searches** |
| --- | --- |
| 1 | Respiratory Distress Syndrome, Adult/ |
| 2 | acute respiratory distress syndrome?.mp. |
| 3 | adult respiratory distress syndrome?.mp. |
| 4 | ARDS.mp. |
| 5 | ARDSS.mp. |
| 6 | shock lung.mp. |
| 7 | Acute Chest Syndrome/ |
| 8 | Acute Lung Injury/ |
| 9 | (acute adj1 chest adj1 syndrome?).mp. |
| 10 | exp ventilator-induced lung injury/ |
| 11 | (ventilat* adj1 induced).mp. |
| 12 | (acute adj1 lung? adj1 fail*).mp. |
| 13 | (acute adj1 pulmonary adj1 fail*).mp. |
| 14 | (acute adj1 bronchopulmonary adj1 fail*).mp. |
| 15 | (acute adj1 respirat* adj1 fail*).mp. |
| 16 | (acute adj1 lung? adj1 injur*).mp. |
| 17 | (acute adj1 pulmonary adj1 injur*).mp. |
| 18 | (acute adj1 bronchopulmonary adj1 injur*).mp. |
| 19 | (acute adj1 respirat* adj1 injur*).mp. |
| 20 | Respiratory Insufficiency/ |
| 21 | (respirat* adj2 insufficien*).mp. |
| 22 | Pulmonary Atelectasis/ |
| 23 | (pulmonary adj2 atelectas*).mp. |
| 24 | (pulmon* adj2 insufficien*).mp. |
| 25 | (bronchopulmon* adj2 insufficien*).mp. |
| 26 | (cardiopulmon* adj2 insufficien*).mp. |
| 27 | ALI.ti,ab. and (lung? or pulmonary or respiratory).mp. [ Acute Lung Injury ] |
| 28 | or/1-27 [ ARDS & related terms ] |
| 29 | exp Positive-Pressure Respiration/ |
| 30 | (driving adj8 pressur*).mp,kw. |
| 31 | (high* adj4 pressur*).mp,kw. |
| 32 | (less*5 adj4 pressur*).mp,kw. |
| 33 | (low*5 adj4 pressur*).mp,kw. |
| 34 | (differen* adj4 pressur*).mp,kw. |
| 35 | (variab* adj4 pressur*).mp,kw. |
| 36 | (varying adj4 pressur*).mp,kw. |
| 37 | Pressure/ and (lung? or pulmonary or respiratory or respiration).mp,kw. |
| 38 | Lung Compliance/ |
| 39 | (lung? adj1 complianc*).mp,kw. |
| 40 | (peak adj1 pressure?).mp,kw. |
| 41 | (plateau adj1 pressure?).mp,kw. |
| 42 | (PEEP*4 and (lung? or pulmonary or respiratory or respiration)).mp,kw. |
| 43 | (positive adj4 end-expiratory pressure?).mp,kw. |
| 44 | (positive adj4 end-expiratory).mp,kw. |
| 45 | (positive adj4 endexpiratory).mp,kw. |
| 46 | end-expiratory pressure?.mp,kw. |
| 47 | (end-expiratory adj4 pressure?).mp,kw. |
| 48 | (open adj2 lung? adj2 strateg*).mp,kw. |
| 49 | (open adj2 lung? adj2 approach*).mp,kw. |
| 50 | (recruit* and (maneuver* or manoeuvr*)).mp,kw. |
| 51 | (lung? adj10 recruit*).mp,kw. |
| 52 | (pulmonary adj10 recruit*).mp,kw. |
| 53 | (respiratory adj10 recruit*).mp,kw. |
| 54 | respiratory system compliance.mp. |
| 55 | Manometry/ and Esophagus/ |
| 56 | (esophageal adj2 manometr*).mp. |
| 57 | or/29-56 [ PEEP & related terms ] |
| 58 | 28 and 57 [ ARDS + PEEP ] |
| 59 | Adaptive Clinical Trial/ |
| 60 | Adaptive Clinical Trials as Topic/ |
| 61 | Clinical Trial, Phase I/ |
| 62 | Clinical Trial, Phase Ii/ |
| 63 | Clinical Trial, Phase Iii/ |
| 64 | Clinical Trial, Phase Iv/ |
| 65 | Clinical Trials, Phase I as Topic/ |
| 66 | Clinical Trials, Phase Ii as Topic/ |
| 67 | Clinical Trials, Phase Iii as Topic/ |
| 68 | Clinical Trials, Phase Iv as Topic/ |
| 69 | Controlled Clinical Trial/ |
| 70 | Controlled Clinical Trials as Topic/ |
| 71 | Double-Blind Method/ |
| 72 | Multicenter Studies as Topic/ |
| 73 | Multicenter Study/ |
| 74 | Placebos/ |
| 75 | Pragmatic Clinical Trial/ |
| 76 | Pragmatic Clinical Trials as Topic/ |
| 77 | Randomized Controlled Trial/ |
| 78 | Randomized Controlled Trials as Topic/ |
| 79 | (("phase 2" or "phase2" or "phase II") and (trial? or study or studies)).mp. |
| 80 | (("phase 3" or "phase3" or "phase III") and (trial? or study or studies)).mp. |
| 81 | (("phase 4" or "phase4" or "phase IV") and (trial? or study or studies)).mp. |
| 82 | ((single or double or triple or treble) adj3 (blind* or mask*)).mp. |
| 83 | (conceal* adj2 allocat*).mp. |
| 84 | (controlled adj1 clinical adj2 (trial? or study or studies)).mp. |
| 85 | (pragmatic adj2 (trial? or study or studies)).mp. |
| 86 | cohort?.mp. |
| 87 | placebo*.mp. |
| 88 | quasirandom*.mp. |
| 89 | randomi*.mp. |
| 90 | semiquantitative.mp. |
| 91 | or/59-90 [ Randomized Controlled Trials & related terms ] |
| 92 | 58 and 91 [ ARDS + PEEP + RCTs ] |
| 93 | exp animals/ not (exp animals/ and humans/) |
| 94 | 92 not 93 |
| 95 | limit 92 to humans |
| 96 | 94 or 95 |
| 97 | limit 96 to ("all infant (birth to 23 months)" or "all child (0 to 18 years)" or "newborn infant (birth to 1 month)" or "infant (1 to 23 months)" or "preschool child (2 to 5 years)" or "child (6 to 12 years)" or "adolescent (13 to 18 years)") |
| 98 | limit 96 to children |
| 99 | 97 or 98 |
| 100 | 96 not 99 |
| 101 | limit 96 to ("all adult (19 plus years)" or "young adult (19 to 24 years)" or "adult (19 to 44 years)" or "young adult and adult (19-24 and 19-44)" or "middle age (45 to 64 years)" or "middle aged (45 plus years)" or "all aged (65 and over)" or "aged (80 and over)") |
| 102 | 100 or 101 |
| 103 | remove duplicates from 102 |

2) Medline In-Process (Ovid MEDLINE(R) Epub Ahead of Print and In-Process & Other Non-Indexed Citations)

| **#** | **Searches** |
| --- | --- |
| 1 | Respiratory Distress Syndrome, Adult/ |
| 2 | acute respiratory distress syndrome?.mp. |
| 3 | adult respiratory distress syndrome?.mp. |
| 4 | ARDS.mp. |
| 5 | ARDSS.mp. |
| 6 | shock lung.mp. |
| 7 | Acute Chest Syndrome/ |
| 8 | Acute Lung Injury/ |
| 9 | (acute adj1 chest adj1 syndrome?).mp. |
| 10 | exp ventilator-induced lung injury/ |
| 11 | (ventilat* adj1 induced).mp. |
| 12 | (acute adj1 lung? adj1 fail*).mp. |
| 13 | (acute adj1 pulmonary adj1 fail*).mp. |
| 14 | (acute adj1 bronchopulmonary adj1 fail*).mp. |
| 15 | (acute adj1 respirat* adj1 fail*).mp. |
| 16 | (acute adj1 lung? adj1 injur*).mp. |
| 17 | (acute adj1 pulmonary adj1 injur*).mp. |
| 18 | (acute adj1 bronchopulmonary adj1 injur*).mp. |
| 19 | (acute adj1 respirat* adj1 injur*).mp. |
| 20 | Respiratory Insufficiency/ |
| 21 | (respirat* adj2 insufficien*).mp. |
| 22 | Pulmonary Atelectasis/ |
| 23 | (pulmonary adj2 atelectas*).mp. |
| 24 | (pulmon* adj2 insufficien*).mp. |
| 25 | (bronchopulmon* adj2 insufficien*).mp. |
| 26 | (cardiopulmon* adj2 insufficien*).mp. |
| 27 | ALI.ti,ab. and (lung? or pulmonary or respiratory).mp. [ Acute Lung Injury ] |
| 28 | or/1-27 [ ARDS & related terms ] |
| 29 | exp Positive-Pressure Respiration/ |
| 30 | (driving adj8 pressur*).mp,kw. |
| 31 | (high* adj4 pressur*).mp,kw. |
| 32 | (less*5 adj4 pressur*).mp,kw. |
| 33 | (low*5 adj4 pressur*).mp,kw. |
| 34 | (differen* adj4 pressur*).mp,kw. |
| 35 | (variab* adj4 pressur*).mp,kw. |
| 36 | (varying adj4 pressur*).mp,kw. |
| 37 | Pressure/ and (lung? or pulmonary or respiratory or respiration).mp,kw. |
| 38 | Lung Compliance/ |
| 39 | (lung? adj1 complianc*).mp,kw. |
| 40 | (peak adj1 pressure?).mp,kw. |
| 41 | (plateau adj1 pressure?).mp,kw. |
| 42 | (PEEP*4 and (lung? or pulmonary or respiratory or respiration)).mp,kw. |
| 43 | (positive adj4 end-expiratory pressure?).mp,kw. |
| 44 | (positive adj4 end-expiratory).mp,kw. |
| 45 | (positive adj4 endexpiratory).mp,kw. |
| 46 | end-expiratory pressure?.mp,kw. |
| 47 | (end-expiratory adj4 pressure?).mp,kw. |
| 48 | (open adj2 lung? adj2 strateg*).mp,kw. |
| 49 | (open adj2 lung? adj2 approach*).mp,kw. |
| 50 | (recruit* and (maneuver* or manoeuvr*)).mp,kw. |
| 51 | (lung? adj10 recruit*).mp,kw. |
| 52 | (pulmonary adj10 recruit*).mp,kw. |
| 53 | (respiratory adj10 recruit*).mp,kw. |
| 54 | respiratory system compliance.mp. |
| 55 | Manometry/ and Esophagus/ |
| 56 | (esophageal adj2 manometr*).mp. |
| 57 | or/29-56 [ PEEP & related terms ] |
| 58 | 28 and 57 [ ARDS + PEEP ] |
| 59 | Adaptive Clinical Trial/ |
| 60 | Adaptive Clinical Trials as Topic/ |
| 61 | Clinical Trial, Phase I/ |
| 62 | Clinical Trial, Phase Ii/ |
| 63 | Clinical Trial, Phase Iii/ |
| 64 | Clinical Trial, Phase Iv/ |
| 65 | Clinical Trials, Phase I as Topic/ |
| 66 | Clinical Trials, Phase Ii as Topic/ |
| 67 | Clinical Trials, Phase Iii as Topic/ |
| 68 | Clinical Trials, Phase Iv as Topic/ |
| 69 | Controlled Clinical Trial/ |
| 70 | Controlled Clinical Trials as Topic/ |
| 71 | Double-Blind Method/ |
| 72 | Multicenter Studies as Topic/ |
| 73 | Multicenter Study/ |
| 74 | Placebos/ |
| 75 | Pragmatic Clinical Trial/ |
| 76 | Pragmatic Clinical Trials as Topic/ |
| 77 | Randomized Controlled Trial/ |
| 78 | Randomized Controlled Trials as Topic/ |
| 79 | (("phase 2" or "phase2" or "phase II") and (trial? or study or studies)).mp. |
| 80 | (("phase 3" or "phase3" or "phase III") and (trial? or study or studies)).mp. |
| 81 | (("phase 4" or "phase4" or "phase IV") and (trial? or study or studies)).mp. |
| 82 | ((single or double or triple or treble) adj3 (blind* or mask*)).mp. |
| 83 | (conceal* adj2 allocat*).mp. |
| 84 | (controlled adj1 clinical adj2 (trial? or study or studies)).mp. |
| 85 | (pragmatic adj2 (trial? or study or studies)).mp. |
| 86 | cohort?.mp. |
| 87 | placebo*.mp. |
| 88 | quasirandom*.mp. |
| 89 | randomi*.mp. |
| 90 | semiquantitative.mp. |
| 91 | or/59-90 [ Randomized Controlled Trials & related terms ] |
| 92 | 58 and 91 [ ARDS + PEEP + RCTs ] |
| 93 | (animal or animals or ape or apes or baboon or baboons or bat or bats or bird or birds or boar or boars or bonobo or bonobos or bovine or camel or camels or canine or canines or cat or cats or cattle or chicken or chickens or chimpanzee or chimpanzees or dog or dogs or dromedary or dromedaries or duck or ducks or equine or equines or feline or felines or ferret or ferrets or frog or frogs or fowl or fowls or goat or goats or hare or hares or hen or hens or horse or horses or lamb or lambs or livestock or macaque or macaques or mandrill or mandrills or mice or mink or minks or monkey or monkeys or mouse or murine or pig or pigs or piglet or piglets or poultry or porcine or orangutan or orangutans or rabbit or rabbits or rat or rats or rodent or rodents or sheep or swine or tamarin or tamarins or tiger or tigers or veterinary or veterinarian or veterinarians or waterfowl or waterfowls or weasel or weasels or veterinar*).ti. or (veterinar* or fish or shellfish).jw. |
| 94 | 92 not 93 |
| 95 | (adolescence or adolescent or adolescents or babies or baby or boy or boys or child or childhood or children or childrens or children's or fetus or fetal or foetus or foetal or girl or girls or infancy or infant or infants or neonatal or neonatally or neonate or neonates or newborn or newborns or paediatric or paediatrician or paediatricians or paediatrics or pediatric or pediatrician or pediatricians or pediatrics or teen or teenage or teenagers or teens or toddler or toddlers or youth or youths).ti,jw. |
| 96 | 94 not 95 |
| 97 | (elder* or senior? or aged or adult*).ti. |
| 98 | 94 and 97 |
| 99 | 96 or 98 |
| 100 | remove duplicates from 99 |

3) Embase (Embase Classic + Embase)

| **#** | **Searches** |
| --- | --- |
| 1 | Respiratory Distress Syndrome, Adult/ |
| 2 | acute respiratory distress syndrome?.mp. |
| 3 | adult respiratory distress syndrome?.mp. |
| 4 | ARDS.mp. |
| 5 | ARDSS.mp. |
| 6 | ARDSnet.mp. |
| 7 | shock lung.mp. |
| 8 | Acute Chest Syndrome/ [New MeSH as of 2010; related to ARDS] |
| 9 | Acute Lung Injury/ |
| 10 | (acute adj1 chest adj1 syndrome?).mp. |
| 11 | exp ventilator-induced lung injury/ |
| 12 | (ventilat* adj1 induced).mp. |
| 13 | (acute adj1 lung? adj1 fail*).mp. |
| 14 | (acute adj1 pulmonary adj1 fail*).mp. |
| 15 | (acute adj1 bronchopulmonary adj1 fail*).mp. |
| 16 | (acute adj1 respirat* adj1 fail*).mp. |
| 17 | (acute adj1 lung? adj1 injur*).mp. |
| 18 | (acute adj1 pulmonary adj1 injur*).mp. |
| 19 | (acute adj1 bronchopulmonary adj1 injur*).mp. |
| 20 | (acute adj1 respirat* adj1 injur*).mp. |
| 21 | Respiratory Insufficiency/ |
| 22 | (respirat* adj2 insufficien*).mp. |
| 23 | Pulmonary Atelectasis/ |
| 24 | (pulmonary adj2 atelectas*).mp. |
| 25 | (pulmon* adj2 insufficien*).mp. |
| 26 | (bronchopulmon* adj2 insufficien*).mp. |
| 27 | (cardiopulmon* adj2 insufficien*).mp. |
| 28 | adult respiratory distress syndrome/ [Embase] |
| 29 | respiratory distress syndrome/ [Embase] |
| 30 | acute chest syndrome/ [Embase] |
| 31 | acute respiratory failure/ [Embase] |
| 32 | respiratory failure/ [Embase] |
| 33 | cardiopulmonary insufficiency/ [Embase] |
| 34 | ALI.ti,ab. [ Acute Lung Injury ] |
| 35 | lung insufficiency/ |
| 36 | or/1-35 [ ARDS ] |
| 37 | positive end expiratory pressure/ |
| 38 | positive end expiratory pressure ventilator/ |
| 39 | lung compliance/ |
| 40 | esophagus manometry/ |
| 41 | Pressure/ and (lung? or pulmonary or respiratory or respiration).mp,kw. |
| 42 | (driving adj8 pressur*).mp,kw. |
| 43 | (high* adj4 pressur*).mp,kw. |
| 44 | (less*5 adj4 pressur*).mp,kw. |
| 45 | (low*5 adj4 pressur*).mp,kw. |
| 46 | (differen* adj4 pressur*).mp,kw. |
| 47 | (variab* adj4 pressur*).mp,kw. |
| 48 | (varying adj4 pressur*).mp,kw. |
| 49 | (lung? adj1 complianc*).mp,kw. |
| 50 | (peak adj1 pressure?).mp,kw. |
| 51 | (plateau adj1 pressure?).mp,kw. |
| 52 | (PEEP*4 and (lung? or pulmonary or respiratory or respiration)).mp,kw. |
| 53 | (positive adj4 end-expiratory pressure?).mp,kw. |
| 54 | (positive adj4 end-expiratory).mp,kw. |
| 55 | (positive adj4 endexpiratory).mp,kw. |
| 56 | end-expiratory pressure?.mp,kw. |
| 57 | (end-expiratory adj4 pressure?).mp,kw. |
| 58 | (open adj2 lung? adj2 strateg*).mp,kw. |
| 59 | (open adj2 lung? adj2 approach*).mp,kw. |
| 60 | (recruit* and (maneuver* or manoeuvr*)).mp,kw. |
| 61 | (lung? adj10 recruit*).mp,kw. |
| 62 | (pulmonary adj10 recruit*).mp,kw. |
| 63 | (respiratory adj10 recruit*).mp,kw. |
| 64 | respiratory system compliance.mp. |
| 65 | (esophageal adj2 manometr*).mp. |
| 66 | or/37-65 [ PEEP & related terms ] |
| 67 | 36 and 66 [ ARDS + PEEP ] |
| 68 | Adaptive Clinical Trial/ |
| 69 | "adaptive clinical trial (topic)"/ |
| 70 | Adaptive Clinical Trials as Topic/ |
| 71 | ct.fs. [ Clinical Trials floating subheading in Embase ] |
| 72 | phase 1 clinical trial/ or "phase 1 clinical trial (topic)"/ or phase 2 clinical trial/ or "phase 2 clinical trial (topic)"/ or phase 3 clinical trial/ or "phase 3 clinical trial (topic)"/ or phase 4 clinical trial/ or "phase 4 clinical trial (topic)"/ |
| 73 | controlled clinical trial/ or "controlled clinical trial (topic)"/ |
| 74 | double blind procedure/ |
| 75 | Double-Blind Method/ |
| 76 | multicenter study/ or "multicenter study (topic)"/ |
| 77 | placebo/ or placebo effect/ |
| 78 | pragmatic trial/ |
| 79 | exp randomized controlled trial/ or "randomized controlled trial (topic)"/ |
| 80 | randomization/ |
| 81 | (("phase 2" or "phase2" or "phase II") and (trial? or study or studies)).mp. |
| 82 | (("phase 3" or "phase3" or "phase III") and (trial? or study or studies)).mp. |
| 83 | (("phase 4" or "phase4" or "phase IV") and (trial? or study or studies)).mp. |
| 84 | ((single or double or triple or treble) adj3 (blind* or mask*)).mp. |
| 85 | (conceal* adj2 allocat*).mp. |
| 86 | (controlled adj1 clinical adj2 (trial? or study or studies)).mp. |
| 87 | (pragmatic adj2 (trial? or study or studies)).mp. |
| 88 | cohort?.mp. |
| 89 | placebo*.mp. |
| 90 | quasirandom*.mp. |
| 91 | quasi-random*.mp. |
| 92 | random*.mp. |
| 93 | semiquantitative.mp. |
| 94 | semi-quantitative.mp. |
| 95 | or/68-94 [ RCTs & related terms ] |
| 96 | 67 and 95 [ ARDS + PEEP + Studies ] |
| 97 | (exp animals/ or exp animal experimentation/ or nonhuman/) not ((exp animals/ or exp animal experimentation/ or nonhuman/) and exp human/) |
| 98 | (animal or animals or ape or apes or baboon or baboons or bat or bats or bird or birds or boar or boars or bonobo or bonobos or bovine or camel or camels or canine or canines or cat or cats or cattle or chicken or chickens or chimpanzee or chimpanzees or dog or dogs or dromedary or dromedaries or duck or ducks or equine or equines or feline or felines or ferret or ferrets or frog or frogs or fowl or fowls or goat or goats or hare or hares or hen or hens or horse or horses or lamb or lambs or livestock or macaque or macaques or mandrill or mandrills or mice or mink or minks or monkey or monkeys or mouse or murine or pig or pigs or piglet or piglets or poultry or porcine or orangutan or orangutans or rabbit or rabbits or rat or rats or rodent or rodents or sheep or swine or tamarin or tamarins or tiger or tigers or veterinary or veterinarian or veterinarians or waterfowl or waterfowls or weasel or weasels or veterinar*).ti. or (veterinar* or fish or shellfish).jw. |
| 99 | 96 not (97 or 98) |
| 100 | limit 96 to human |
| 101 | 99 or 100 |
| 102 | limit 101 to (embryo <first trimester> or infant <to one year> or child <unspecified age> or preschool child <1 to 6 years> or school child <7 to 12 years> or adolescent <13 to 17 years>) |
| 103 | 101 not 102 |
| 104 | limit 101 to (adult <18 to 64 years> or aged <65+ years>) |
| 105 | 103 or 104 |
| 106 | limit 105 to (conference abstracts or (books or chapter or conference abstract or "conference review") or (book or book series or conference proceeding)) |
| 107 | 105 not 106 |
| 108 | remove duplicates from 107 |

4) CCTR (Cochrane Central Register of Controlled Trial)

| **#** | **Searches** |
| --- | --- |
| 1 | Respiratory Distress Syndrome, Adult/ |
| 2 | acute respiratory distress syndrome?.mp. |
| 3 | adult respiratory distress syndrome?.mp. |
| 4 | ARDS.mp. |
| 5 | ARDSS.mp. |
| 6 | ARDSnet.mp. |
| 7 | shock lung.mp. |
| 8 | Acute Chest Syndrome/ [New MeSH as of 2010; related to ARDS] |
| 9 | Acute Lung Injury/ |
| 10 | (acute adj1 chest adj1 syndrome?).mp. |
| 11 | exp ventilator-induced lung injury/ |
| 12 | (ventilat* adj1 induced).mp. |
| 13 | (acute adj1 lung? adj1 fail*).mp. |
| 14 | (acute adj1 pulmonary adj1 fail*).mp. |
| 15 | (acute adj1 bronchopulmonary adj1 fail*).mp. |
| 16 | (acute adj1 respirat* adj1 fail*).mp. |
| 17 | (acute adj1 lung? adj1 injur*).mp. |
| 18 | (acute adj1 pulmonary adj1 injur*).mp. |
| 19 | (acute adj1 bronchopulmonary adj1 injur*).mp. |
| 20 | (acute adj1 respirat* adj1 injur*).mp. |
| 21 | Respiratory Insufficiency/ |
| 22 | (respirat* adj2 insufficien*).mp. |
| 23 | Pulmonary Atelectasis/ |
| 24 | (pulmonary adj2 atelectas*).mp. |
| 25 | (pulmon* adj2 insufficien*).mp. |
| 26 | (bronchopulmon* adj2 insufficien*).mp. |
| 27 | (cardiopulmon* adj2 insufficien*).mp. |
| 28 | adult respiratory distress syndrome/ [Embase] |
| 29 | respiratory distress syndrome/ [Embase] |
| 30 | acute chest syndrome/ [Embase] |
| 31 | acute respiratory failure/ [Embase] |
| 32 | respiratory failure/ [Embase] |
| 33 | cardiopulmonary insufficiency/ [Embase] |
| 34 | ALI.ti,ab. [ Acute Lung Injury ] |
| 35 | lung insufficiency/ |
| 36 | or/1-35 [ ARDS ] |
| 37 | positive end expiratory pressure/ |
| 38 | positive end expiratory pressure ventilator/ |
| 39 | lung compliance/ |
| 40 | esophagus manometry/ |
| 41 | Pressure/ and (lung? or pulmonary or respiratory or respiration).mp,kw. |
| 42 | (driving adj8 pressur*).mp,kw. |
| 43 | (high* adj4 pressur*).mp,kw. |
| 44 | (less*5 adj4 pressur*).mp,kw. |
| 45 | (low*5 adj4 pressur*).mp,kw. |
| 46 | (differen* adj4 pressur*).mp,kw. |
| 47 | (variab* adj4 pressur*).mp,kw. |
| 48 | (varying adj4 pressur*).mp,kw. |
| 49 | (lung? adj1 complianc*).mp,kw. |
| 50 | (peak adj1 pressure?).mp,kw. |
| 51 | (plateau adj1 pressure?).mp,kw. |
| 52 | (PEEP*4 and (lung? or pulmonary or respiratory or respiration)).mp,kw. |
| 53 | (positive adj4 end-expiratory pressure?).mp,kw. |
| 54 | (positive adj4 end-expiratory).mp,kw. |
| 55 | (positive adj4 endexpiratory).mp,kw. |
| 56 | end-expiratory pressure?.mp,kw. |
| 57 | (end-expiratory adj4 pressure?).mp,kw. |
| 58 | (open adj2 lung? adj2 strateg*).mp,kw. |
| 59 | (open adj2 lung? adj2 approach*).mp,kw. |
| 60 | (recruit* and (maneuver* or manoeuvr*)).mp,kw. |
| 61 | (lung? adj10 recruit*).mp,kw. |
| 62 | (pulmonary adj10 recruit*).mp,kw. |
| 63 | (respiratory adj10 recruit*).mp,kw. |
| 64 | respiratory system compliance.mp. |
| 65 | (esophageal adj2 manometr*).mp. |
| 66 | or/37-65 [ PEEP & related terms ] |
| 67 | 36 and 66 [ ARDS + PEEP ] |
| 68 | (animal or animals or ape or apes or baboon or baboons or bat or bats or bird or birds or boar or boars or bonobo or bonobos or bovine or camel or camels or canine or canines or cat or cats or cattle or chicken or chickens or chimpanzee or chimpanzees or dog or dogs or dromedary or dromedaries or duck or ducks or equine or equines or feline or felines or ferret or ferrets or frog or frogs or fowl or fowls or goat or goats or hare or hares or hen or hens or horse or horses or lamb or lambs or livestock or macaque or macaques or mandrill or mandrills or mice or mink or minks or monkey or monkeys or mouse or murine or pig or pigs or piglet or piglets or poultry or porcine or orangutan or orangutans or rabbit or rabbits or rat or rats or rodent or rodents or sheep or swine or tamarin or tamarins or tiger or tigers or veterinary or veterinarian or veterinarians or waterfowl or waterfowls or weasel or weasels or veterinar*).ti. or (veterinar* or fish or shellfish).jw. |
| 69 | 67 not 68 |
| 70 | (adolescence or adolescent or adolescents or babies or baby or boy or boys or child or childhood or children or childrens or children's or fetus or fetal or foetus or foetal or girl or girls or infancy or infant or infants or neonatal or neonatally or neonate or neonates or newborn or newborns or paediatric or paediatrician or paediatricians or paediatrics or pediatric or pediatrician or pediatricians or pediatrics or teen or teenage or teenagers or teens or toddler or toddlers or youth or youths).ti,jw. |
| 71 | 69 not 70 |
| 72 | (elder* or senior? or aged or adult*).ti,jw. |
| 73 | 69 and 72 |
| 74 | 71 or 73 |
| 75 | (book or book article or book book or book note or "book review" or book series article or book series article in press or book series chapter or book series conference paper or book series letter or "book series review" or book series short survey or chapter or conference abstract or conference abstract placebo controlled partly blinded crossover study in 12 sle patients or conference proceeding or "conference review" or journal conference abstract or "journal conference review").pt. |
| 76 | conferenc*.so. |
| 77 | presentation*.so. |
| 78 | exhibition*.so. |
| 79 | poster?.so. |
| 80 | or/75-79 |
| 81 | 74 not 80 |
| 82 | limit 81 to (medline records or embase records) |
| 83 | 81 not 82 |
| 84 | remove duplicates from 83 |
